# Efficacy of Mouth Rinses and Nasal Spray in the Inactivation of SARS-CoV-2: A Systematic Review and Meta-Analysis of *In Vitro* and *In Vivo* Studies

**DOI:** 10.3390/ijerph191912148

**Published:** 2022-09-25

**Authors:** Majdy Idrees, Bridget McGowan, Amr Fawzy, Abdulwahab Ali Abuderman, Ramesh Balasubramaniam, Omar Kujan

**Affiliations:** 1 UWA Dental School, The University of Western Australia, Nedlands, WA 6009, Australia; 2 Private Dental Practice, Darwin, NT 0810, Australia; 3 College of Medicine, Prince Sattam bin Abdulaziz University, Alkharj 11942, Saudi Arabia

**Keywords:** SARS-CoV-2, mouth rinse, nasal spray, viral load, meta-analysis

## Abstract

Severe acute respiratory syndrome-coronavirus-2 (SARS-CoV-2) is a global and evolving pandemic associated with heavy health and financial burdens. Considering the oral cavity as the major reservoir for SARS-CoV-2, a systematic review and meta-analysis were conducted to assess the efficacy of mouth rinses and nasal sprays in reducing the salivary viral load of SARS-CoV-2. All *in vivo* and *in vitro* studies that assessed the virucidal efficacy of mouth rinses and nasal sprays against SARS-CoV-2 and were published in the English language from December 2019 to April 2022 were considered for analyses. Special Medical Subject Headings terms were used to search Pubmed, Scopus, Embase Ovid, and Web of Science databases. The toxicological data reliability assessment tool (ToxRToool) was used to assess the quality of the included studies. Thirty-three studies (11 *in vivo* and 22 *in vitro*) were deemed eligible for inclusion in this analysis. Results of the pooled data showed that povidone-iodine is the most efficacious intervention *in vivo* in terms of reducing the SARS-CoV-2 salivary viral load, followed by chlorhexidine. The mean difference in the viral load was 86% and 72%, respectively. Similarly, povidone-iodine was associated with the highest log_10_ reduction value (LRV) *in vitro*, followed by cetylpyridinium chloride, (LRV = 2.938 (*p* < 0.0005) and LRV = 2.907 (*p* = 0.009), respectively). Povidone-iodine-based oral and nasal preparations showed favourable results in terms of reducing SARS-CoV-2 viral loads both *in vivo* and *in vitro*. Considering the limited number of patients *in vivo*, further studies among larger cohorts are recommended.

## 1. Introduction

The coronavirus disease 2019 (COVID-19) pandemic, caused by the severe acute respiratory syndrome coronavirus 2 (SARS-CoV-2), instigated a global health emergency since its discovery in Wuhan, Hubei Provence, China, in 2019 [1]. This virus is a betacoronavirus associated with symptoms ranging from mild respiratory symptoms to severe pneumonia in the lungs, requiring supplementary oxygen or ventilation [1].

The main modes of transmission include human-to-human transmission through droplets, contact with an infected individual, or direct or indirect contact with contaminated surfaces [2]. SARS-CoV-2 can also be transmitted via aerosols, which are defined as air-borne suspended particles with the potential to contain salivary components and microorganisms [3]. Studies have demonstrated a high viral load in the saliva and oropharynx of symptomatic and asymptomatic patients with COVID-19 [4].

Considering that the oral cavity represents the major reservoir for SARS-CoV-2 [4], dental professionals are at high risk of exposure to pathogens through exposure to secretions, inhalation during aerosol-generating procedures, and mucosal contact with infected particles [5]. SARS-CoV-2 has demonstrated the potential for aerosolization for up to 3–16 h *in vitro* studies [6,7]. Thus, many dental regulatory authorities released recommendations to support using pre-procedural mouth rinse in dental settings to reduce SARS-CoV-2 transmission [8,9]. The premise for this is based on the principle of reducing oral microbial loads and mitigating the potential transmission of microbes via aerosol.

In this respect, several active compounds against bacteria and viruses in the oral cavity are currently being assessed for their potential virucidal efficacy against SARS-CoV-2. Studies have been conducted predominately *in vitro* and increasingly *in vivo* on the efficacy and efficiency of various active compounds in mouthwashes to reduce viral loads and, thus, the transmission of COVID-19. Several previous reviews attempted to address this topic; however, due to incomplete ongoing studies, the results were inconclusive [10,11,12,13].

This study aims to assess the evidence from studies that utilized mouth rinses or nasal sprays to reduce the salivary viral load of SARS-CoV-2 and provide evidence-based recommendations that can be employed by decision makers and regulatory authorities to aid in limiting the spread of this infection in the community. 

## 2. Materials and Methods

### 2.1. Protocol and Registration

The proposed systematic review and meta-analysis were registered on the International Prospective Register of Systematic Reviews (PROSPERO) platform (CRD42022323586) and performed following the Preferred Reporting Items for Systematic Reviews and Meta-Analysis (PRISMA) [14]. PRISMA checklists are shown in Supplementary Tables S1 and S2. 

### 2.2. Focused Questions

This review was designed to address the following questions: (a) What is the efficacy of various mouth rinse ingredients in reducing the viral load of SARS-CoV-2 *in vitro*? (b) Are mouth rinses and nasal sprays clinically efficacious in reducing the viral load of SARS-CoV-2? (c) What is the most efficacious mouth rinse for reducing the viral load of SARS-CoV-2 in the community?

### 2.3. Eligibility Criteria

*Inclusion criteria:* Observational studies, both *in vivo* and *in vitro* and published in the English language from December 2019 to April 2022 to assess the virucidal efficacy of mouth rinses and/or nasal sprays against SARS-CoV-2, were assessed for inclusion. 

In vivo studies were included if they (1) recruited subjects with confirmed positive SARS-CoV-2 as per the standard quantitative polymerase chain reaction (qPCR) assays; (2) reported individual subject-level reports of SARS-CoV-2 viral load at two time frames: basal level (viral load just before using a mouth rinse or nasal spray) and experimental level (viral load after 1 to 3 h post using a mouth rinse or nasal spray); and (3) reported values of viral load as either copies/mL or cycle threshold values of PCR assay. 

In vitro studies were included if they: (1) assessed the virucidal efficacy of preparations against a strain of SARS-CoV-2; (2) are studies that reported the Log_10_ reduction value (LRV) between control and experimental groups; (3) are studies that followed the European standards for chemical disinfectants and antiseptics (EN 14476:2013+A2:2019) [15].

*Exclusion criteria:* Letters to the editor, author comments, systematic reviews, and books or book chapters were excluded. In vivo studies that used assays other than qPCR to confirm SARS-CoV-2 positivity were excluded. In vitro studies that used virus surrogates instead of SARS-CoV-2 or did not provide detailed methodological designs were excluded.

### 2.4. Search Strategy and Data Extraction

Eligible studies were identified by using a developed search strategy for the following databases: MEDLINE by PubMed, Scopus, Embase Ovid, and Web of Science (Supplementary Table S3). Titles, abstracts, and keywords of retrieved studies were first screened blindly by two reviewers (MI and OK) to assess their relevance. Subsequently, studies that were considered potentially eligible were assessed by the reviewers by reading the entire text against the pre-defined inclusion and exclusion criteria. Only studies that were eligible for inclusion as agreed by the two reviewers were included. Any discrepancy in the assessment was resolved by discussion and consensus. 

Two authors (MI and OK) extracted relevant data from the included studies. For *in vivo* studies, the following details were collected: (1) authors, country of the experiments, and year of publication; (2) active ingredients, concentrations, and route of administration; (3) number of participants; and (4) viral loads vs. time plots in log_10_. Where possible, numerical values were extracted from tables directly. Viral load values that were reported in figures or plots were extracted using a special online tool, WebPlotDigitizer, accessed from https://automeris.io/WebPlotDigitizer/ (accessed on 15 April 2022). Values that were reported as copies/mL or copies/µL were converted to log_10_ copies/µL. Values that were reported as cycle threshold of PCR assays were converted to log_10_ using the proposed model of Gentilini et al. to convert cycle threshold values to log_10_ copies/µL values [16].

For the *in vitro* studies, the following details were extracted: (1) authors and year of publication; (2) active ingredients and concentrations; (3) the strain of SARS-CoV-2 and the used cell line for propagation; (4) number of replicates and *p* values; and (4) the log_10_ reduction value (LRV) between control and experimental groups. 

### 2.5. Risk of Bias Assessment

The quality of the included studies was assessed using the toxicological data reliability assessment tool (ToxRToool) [17]. ToxRTool includes a specific part for each *in vivo* and *in vitro* study. Slight modifications were made to meet the nature of the included studies. The included *in vivo* and *in vitro* studies were assessed against 15 and 17 items, respectively (Supplementary Tables S7 and S8). Any study that scored 12 or more was considered “reliable without restrictions” according to the systematic approach of Klimisch et al. for evaluating the quality of experimental toxicological and ecotoxicological data [18].

### 2.6. Data Analysis

The pooled difference in the mean and the 95% confidence interval (CI) of viral load for *in vivo* studies and the difference in the mean of LRV for *in vitro* studies were calculated under the random-effects model of the DerSimonian and Laird method. The extent of heterogeneity was measured according to the Higgins method and reported as I^2^. The value of I^2^ was interpreted as low (0–40%), moderate (30% to 60%), substantial (50% to 90%), and considerable (75% to 100%). A *p*-value of <0.05 was considered statistically significant. Review Manager 5.3 (Copenhagen, Denmark) and Comprehensive Meta-Analysis 3.3.070 (Englewood, NJ, USA) were used for meta-analysis purposes. 

## 3. Results

### 3.1. Results of Database Searches

**Figure 1:**
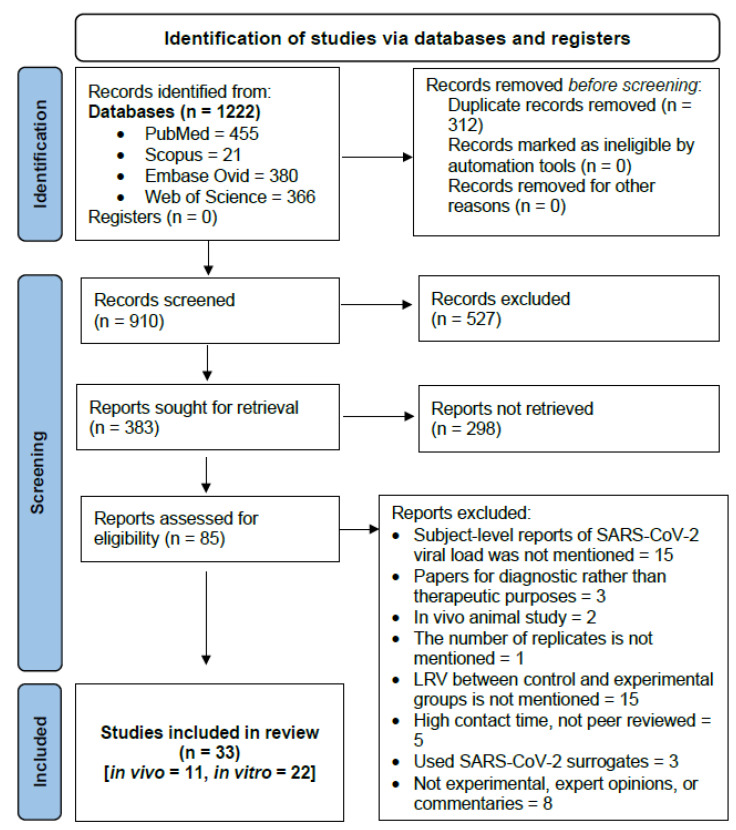
PRISMA flow chart shows the number of screened, included, and excluded studies.

Three hundred and eighty-three studies were retrieved for abstract screening out of one thousand two hundred and twenty-two studies that were initially identified via the search platforms. Of these, 85 studies were assessed by full-text reading, whereby 52 studies were deemed ineligible. Of the included 33 studies, there were 11 *in vivo* studies and 22 *in vitro* studies (Figure 1). Descriptions of the included studies are shown in Supplementary Table S4 for *in vivo* studies and Supplementary Table S5 for *in vitro* studies. The excluded studies and reasons for exclusion are shown in Supplementary Table S6.

### 3.2. General Characteristics of the Included Studies

#### 3.2.1. *In Vivo* Studies

Eleven *in vivo* studies assessed the virucidal efficacies of various preparations among 339 SARS-CoV-2 positive patients [19,20,21,22,23,24,25,26,27,28,29]. Five studies [21,22,24,26,29] conducted several experiments using various preparations, which totaled up to 21 experiments. Only one study assessed the efficacy of nasal sprays in reducing viral load [29] while others used mouth rinses for this purpose. 

Chlorhexidine mouth rinse was assessed in six experiments among 105 patients [20,21,22,24,26,28]. Two experiments assessed chlorhexidine combined with hydrogen peroxide in 23 patients [21,24], while three experiments assessed hydrogen peroxide alone among 28 patients [23,24]. Povidone-iodine was used in five experiments among 55 patients [21,22,25,26,29], and only two experiments utilized cetylpyridinium-chloride mouth rinse in 11 patients [21,26]. Finally, three different preparations were assessed once in three different experiments. These preparations are (1) b-cyclodextrinecitrox mouthwash (CDCM) (76 patients) [19], (2) Linola Sept, Dr August Wolff mouthwash (Linola Sept) (29 patients) [27], and (3) sodium hypochlorite (NaCIO) (12 patients) [24].

Regarding the risk of bias assessment of the *in vivo* studies, all studies were considered reliable without restrictions. However, none of them scored 15 out of 15 as the strain of SARS-CoV-2 was not mentioned in any study (Supplementary Table S7). Seven studies were found to have an overall score of 13, while four studies had a score of 12. 

#### 3.2.2. *In Vitro* Studies

In total, the included *in vitro* studies (22 studies) conducted 79 experiments on different test products at different timescales [30,31,32,33,34,35,36,37,38,39,40,41,42,43,44,45,46,47,48,49,50,51]. The contact time ranged from 15 s to 10 min. Povidone-iodine was the most widely assessed preparation (32 experiments), followed by chlorhexidine and sodium fluoride/chloride in 11 and 9 experiments, respectively (Supplementary Table S5).

In terms of the risk of bias assessment, all studies were reliable for inclusion without restrictions. Four studies had a score of 17 out of 17. Sixteen studies showed an overall score of either 16 or 15. Only one study had a score of 14 (Supplementary Table S8). 

The strain of the assessed virus has varied according to the country of origin. The most widely assessed strain in 11 studies was USA-WA1/2020 [30,31,32,33,34,35,36,37,38,39,40]. The other assessed strains were hCoV19/Singapore/2/2020 [41], England 2 strain [42], SARS-CoV-2/MY/UM/6-3 [43], JPN/TY/WK-521 [44,45], FI-100 strain [46], hCoV-19/ Germany/BY-Bochum-1/2020 [47], and isolate 026 V-03883 [48]. Oropharyngeal swabs from COVID-19-infected patients were used to isolate the virus in two studies [49,50]. All studies propagated the virus into Vero cells (lineages Vero 76 and Vero E6). 

### 3.3. Meta-Analysis for the Virucidal Efficacy of Different Preparations against SARS-CoV-2 In Vivo

**Figure 2:**
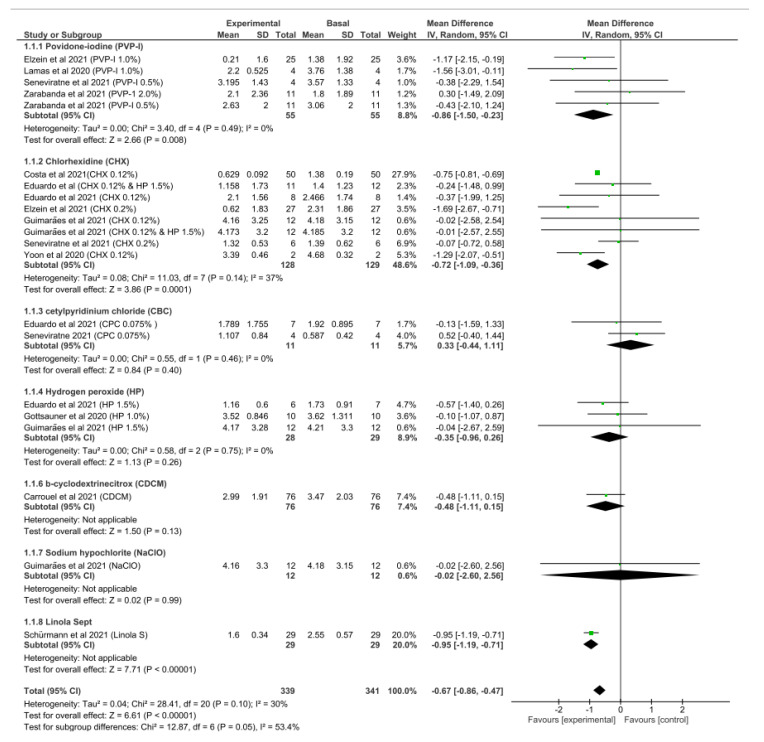
Forest plot of virucidal efficacy of several preparations against SARS-CoV-2 *in vivo* and the associated level of heterogeneity [19,20,21,22,23,24,25,26,27,28,29].

Pooled data revealed that using a mouth rinse or nasal spray *in vivo* significantly reduced the salivary viral load for a period ranging from 5 min to 3 h by 67% (CI 95% −0.86, −0.47) (*p* < 0.0001) (Figure 2). The level of heterogeneity among the experiments was low (I^2^ = 30%); however, this was not statistically significant (*p* = 0.10) (Figure 2). 

For sub-group analysis, pooled data showed that povidone-iodine was associated with the highest virucidal efficacy in comparison to other preparations, with a significant mean difference in the viral load (−0.86 [CI 95% −1.5, −0.23], *p* = 0.008). The second most efficacious preparation *in vivo* was chlorhexidine, which showed a statistically significant reduction in the viral load (−0.72 [CI 95% −1.09, −0.36], *p* = 0.0001) (Figure 2). However, further subgroup analyses for povidone-iodine and chlorhexidine based on their concentrations were not possible due to the limited number of patients in certain concentrations. 

Using cetylpyridinium-chloride and hydrogen peroxide preparations was not associated with a significant reduction in viral load (*p* = 0.4 and *p* = 0.26, respectively) (Figure 2). 

### 3.4. Meta-Analysis for the Virucidal Efficacy of Different Preparations against SARS-CoV-2 In Vitro

**Figure 3:**
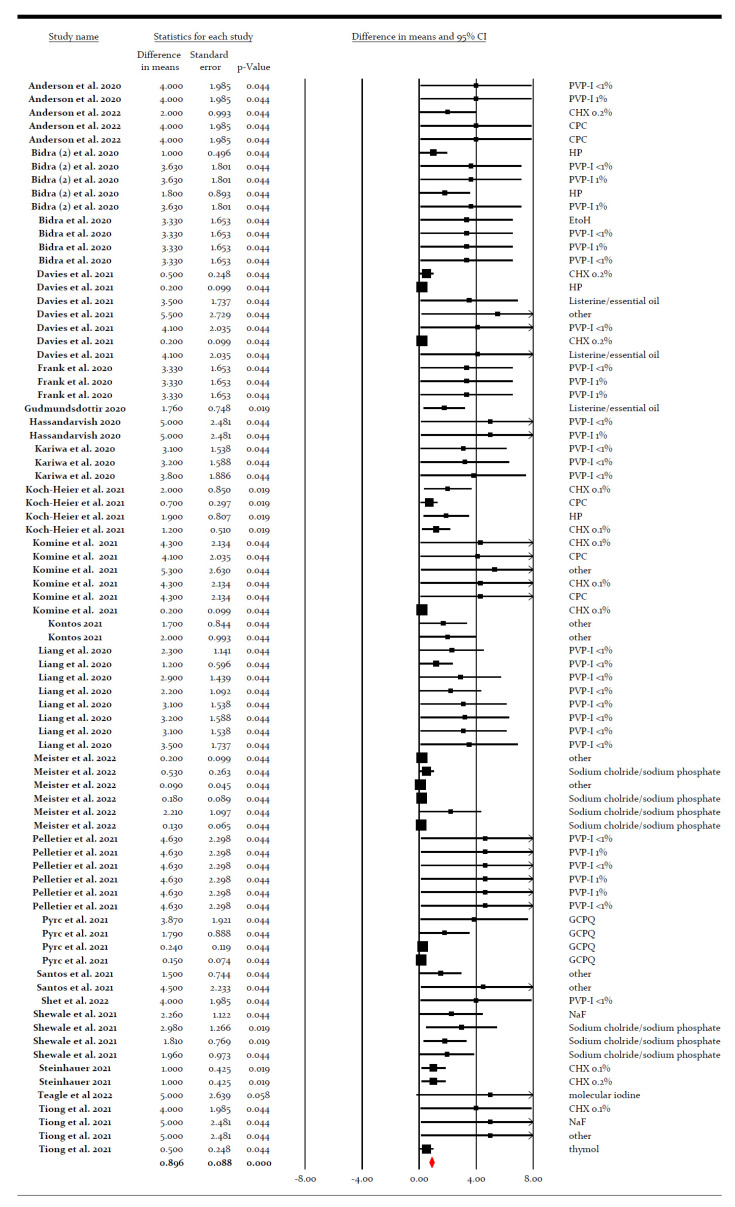
Forest plot of virucidal efficacy of several preparations against SARS-CoV-2 *in vitro* [30,31,32,33,34,35,36,37,38,39,40,41,42,43,44,45,46,47,48,49,50,51].

Pooled data for the entire set of 79 *in vitro* experiments showed an LRV of 0.886 (*p* < 0.0005) (Figure 3). However, there was substantial heterogeneity among the experiments (I^2^ = 69.192, *p* < 0.0005) (Supplementary Figure S1). 

**Figure 4:**
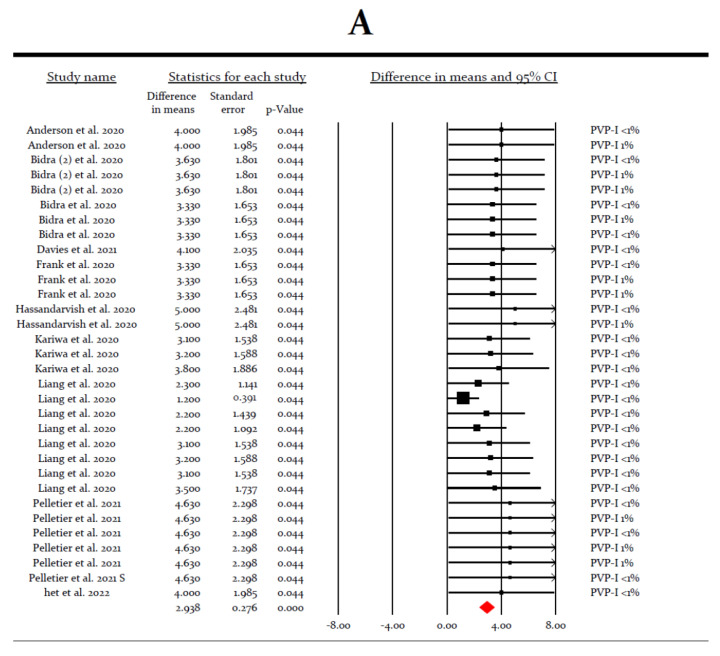

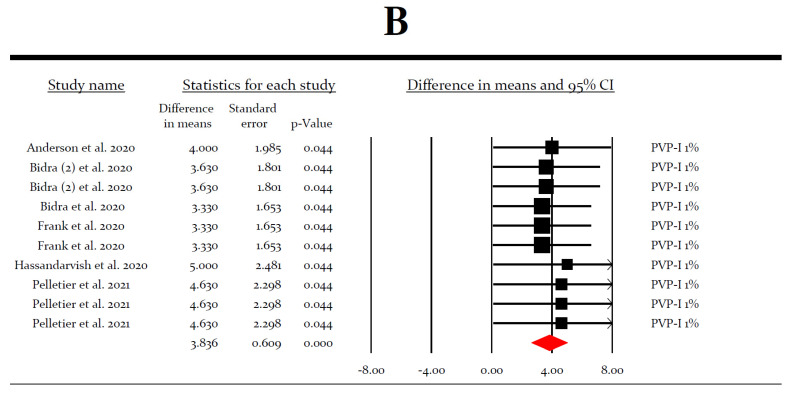
(**A**) Forrest plot of virucidal efficacy of povidone-iodine (PVP-I) (all concentrations) *in vitro* [31,32,33,34,35,36,37,38,41,42,43,45]. (**B**) Forrest plot of virucidal efficacy of povidone-iodine (PVP-I) > 1.0% *in vitro* [31,32,33,37,41,43].

After applying subgroup analyses, the most efficacious intervention was povidone-iodine, regardless of its concentration (LRV = 2.938, *p* < 0.0005) (Figure 4A). Additional subgroup analysis among povidone-iodine experiments showed that the LRV increased to 3.836 (*p* < 0.0005) when using povidone-iodine in concentrations of more than 1% (Figure 4B).

**Figure 5:**
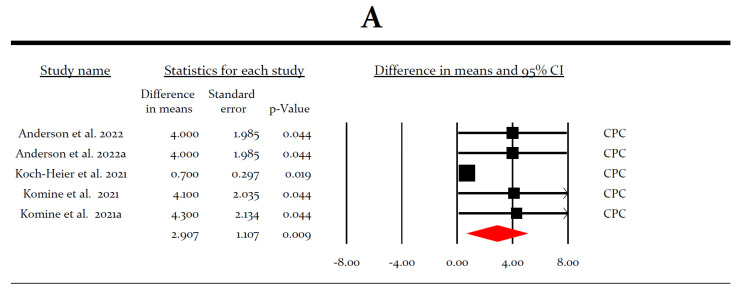

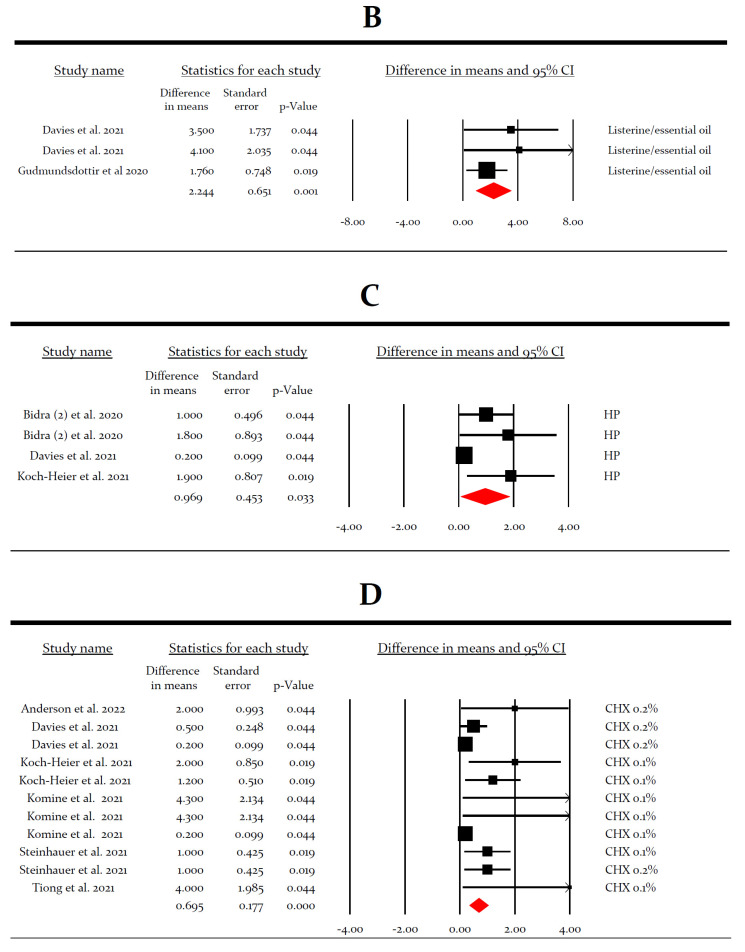

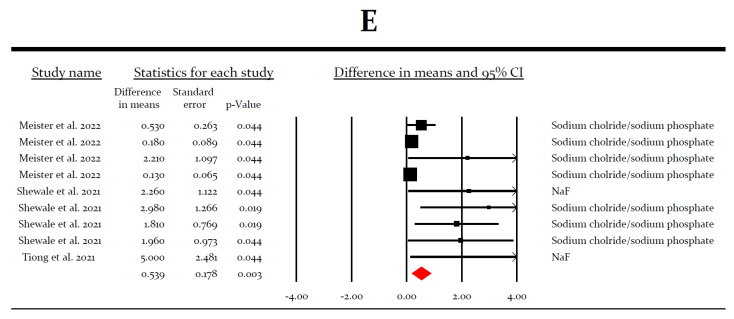
(**A**) Forrest plot of virucidal efficacy of cetylpyridinium-chloride (CPC) *in vitro* [30,44,46]. (**B**) Forrest plot of virucidal efficacy of Listerine^®^ and other essential oils *in vitro* [42,43]. (**C**) Forrest plot of virucidal efficacy of hydrogen peroxide (HP) *in vitro* [32,42,46]. (**D**) Forrest plot of virucidal efficacy of chlorhexidine (CHX) *in vitro* [30,42,44,46,50,51]. (**E**) Forrest plot of virucidal efficacy of sodium fluoride, sodium chloride, and sodium fluoride (NaF) *in vitro* [39,47,50].

The second most efficacious intervention in terms of *in vitro* virucidal activity was cetylpyridinium-chloride with an LRV of 2.907 (*p* = 0.009) (Figure 5A). The third most efficacious intervention was Listerine^®^ and other essential oils (LRV = 2.244, *p* = 0.001) (Figure 5B). 

A statistically significant difference in LRV was reported for hydrogen peroxide (LRV = 0.969, *p* = 0.033) (Figure 5C). Eleven experiments utilizing chlorhexidine and their related pool data revealed an LRV of 0.695 (*p* < 0.0005) (Figure 5D). Finally, the least efficacious intervention was related to preparations containing sodium fluoride or sodium chloride (LRV = 0.539, *p* = 0.003) (Figure 5E).

## 4. Discussion

Since the emergence of the pandemic, viral loads of SARS-CoV-2 have varied greatly among patients and played a significant role in infectivity and fatality. Although many occupational-related organizations recommended using mouth rinses to reduce the SARS-CoV-2 viral load to levels that could be non-infectious [8,9], these recommendations were anecdotal, speculative, and not evidence-based. This systematic review presents an evaluation of the available evidence about the efficacy of mouth rinses and nasal sprays in decreasing the SARS-CoV-2 viral load to potentially prevent the spread of COVID-19 in the community. To the best of our knowledge, this work represents the first meta-analysis for both *in vivo* and *in vitro* studies in this area.

SARS-CoV-2 infection is usually diagnosed qualitatively as positive or negative, although the gold standard for its diagnosis, the quantitative polymerase chain reaction, was originally intended to be quantitative. This greatly hampered the understanding of the viral load’s dynamics and may explain why some individuals are infectious without symptoms, while similarly infectious individuals do not transmit the infection to their intimate partner and household members.

In this review, the majority of *in vivo* studies reported their viral load outcomes as cycle threshold values for qPCR. However, neither methods of cycle threshold conversion to viral load nor measured errors were provided [16]. Such inconsistencies in reports complicate comparisons between studies. To overcome this obstacle, this review used the proposed model of Gentilini et al. in 2021 to convert cycle threshold values to SARS-CoV-2 viral load [16]. This model was validated on more than 6200 COVID-19-positive patients and showed a reliability of almost 92% [16]. The results of this review found that *in vitro* studies followed the European standards for chemical disinfectants and antiseptics (EN14476:2013+A2:2019) [15]. According to these standards, a substance can be considered virucidal efficacious if it reduces the virus titre by at least four decimal logarithms (LRV ≥ 4 log_10_) [15]. However, the analysis of the pooled data showed that all preparations were associated with LRV less than 4 log_10_. This may highlight the potential limitations of *in vitro* studies. SARS-CoV-2 primarily targets the human lung epithelium; nonetheless, the most widely used cell line in SARS-CoV-2-related projects is the Vero cell line (kidney of an African green monkey). This is because Vero cells express high levels of ACE 2, which is the cellular receptor for SARS-CoV-2 entry [52]. Noteworthily, the members of the World Health Organization (WHO) working on the SARS-CoV-2 virus analysed genetic sequencing data and found that the virus propagation in Vero cells causes genetic variants that may impact the interpretation of results from animal and clinical trials [53]. This may, to some extent, explain the differences between *in vivo* and *in vitro* results for some preparations. 

One of the most widely studied preparations is povidone-iodine. Povidone-iodine is a broad-spectrum antimicrobial compound of a potent bactericidal agent, iodine, which is within the carrier molecule, povidone [41]. The results of this meta-analysis revealed that povidone-iodine is the most efficacious virucidal preparation against SARS-CoV-2 in both *in vivo* and *in vitro* studies. The mean difference in viral load was found to be up to 86% for up to 3 h post rinsing with povidone-iodine. While its efficacy increases with concentrations of more than 1%, the time for oral rinses ranges between 1 and 2 min. Considering that povidone-iodine can safely be used in the oral cavity at concentrations up to 2.5% for up to 5 months [22], this indicates the potential efficaciousness of povidone-iodine in controlling the spread of SARS-CoV-2. 

Chlorhexidine is a cationic surfactant and synthetic biguanide with broad-spectrum antimicrobial activity [11]. Although it is well documented that chlorhexidine mouthwash is effective against herpes, influenza, parainfluenza, and hepatitis B [11], the available evidence about its efficacy against SARS-CoV-2 is limited. Our analysis of the *in vivo* studies demonstrated that chlorhexidine is the second most efficacious intervention against SARS-CoV-2 as it lowered the mean oral viral load by 72% (*p* = 0.0001). On the contrary, *in vitro* experiments showed a very limited therapeutic utility for chlorhexidine in reducing the viral load. This can be attributed to the fact that chlorhexidine provides a long-lasting effect *in vivo* due to its ability to retain on oral cavity surfaces for several hours because of its cationic feature, whereas it cannot possess the same antiseptic effect with a short contact time *in vitro* [11].

Hydrogen peroxide is an antiseptic solution that exerts its microbicidal action by producing hydroxyl free radicals that can attack membrane lipids and other essential cell components of pathogens. [24,30]. It has been suggested that using 1% hydrogen peroxide would be more appropriate for reducing the salivary load of SARS-CoV-2 as the virus is vulnerable to oxidation in the oral environment. However, the results of this review revealed that hydrogen peroxide oral rinse is not superior to other preparations in reducing the salivary load of SARS-CoV-2, both *in vivo* and *in vitro* (35% and LRV = 0.969, respectively). 

Cetylpyridinium-chloride is a quaternary ammonium compound that exerts its antiviral effect through a physiochemical disruption of the viral lipid envelope, which is the same as the membrane surrounding SARS-CoV-2 [30]. Although our results revealed that cetylpyridinium-chloride did not show promising results *in vivo*, it was the second most efficacious preparation after povidone-iodine *in vitro* (LRV = 2.907). However, as the number of patients *in vivo* was limited (11 patients), a further validation of cetylpyridinium-chloride in larger cohorts is required to provide reliable recommendations.

The major limitation of this study was the limited number of patients for *in vivo* studies. This can be attributed to strict ethical requirements for *in vivo* studies in some countries. The second major limitation is the difference in the number of studies and participants among *in vivo* studies for some active ingredients, which may make comparisons between the efficacy of these ingredients non-conclusive. However, after more than two years of this pandemic, we anticipate the easing of mandatory restrictions, which will translate to increased quality and quantity of clinical studies. Moreover, the absence of standardized protocols for sample collection and result reporting among *in vivo* studies accounts for heterogeneity among the relevant studies. Finally, all *in vitro* studies utilised Vero cells for the virus propagation; however, based on the recent WHO report, there is sufficient reason to question the reliability of Vero cells in these experiments [53].

## 5. Conclusions

In conclusion, povidone-iodine and chlorhexidine mouth rinses, regardless of concentration, were clinically the most efficacious interventions for reducing the SARS-CoV-2 oral viral load. Emerging evidence from *in vivo* studies using hydrogen peroxide, cetylpyridinium-chloride, and various other active ingredients remains inconclusive. Despite povidone-iodine and chlorhexidine mouth rinses demonstrating favourable efficacy, their effectiveness in terms of virucidal activity does not currently meet recommended standards of the European Standards for chemical disinfectants and antiseptics (EN 14476). Given these results, governing organisations should revisit their COVID-19 pandemic guidelines and consider recommending specific preparations of mouth rinses (1–5% povidone-iodine or 0.12–0.2% chlorhexidine) for individuals infected by SARS-CoV-2 or at a high risk of being infected. However, prescribers should be aware of the side effects of these mouthwashes when they plan to use them routinely. Likewise, consideration for routine use of mouth rinses by asymptomatic or uninfected individuals during high community transmission may reduce the burden on strained health care systems. Nonetheless, new mouthwashes with little to no side effects that can significantly reduce the SARS-CoV-2 oral viral load with remarkable virucidal impact are warranted.

## Data Availability

Data is contained within the article and supplementary materials.

## Supplementary Materials

The following supporting information can be downloaded at: https://www.mdpi.com/article/10.3390/ijerph191912148/s1, Figure S1: Funnel plot of the standard error by the difference in means for *in vitro* studies; Table S1: PRISMA 2020 main checklist; Table S2: PRIMSA 2020 Abstract Checklist; Table S3: MeSH terms used for searching through Pubmed, Scopus, Embase Ovid, and Web of Science databases; Table S4: General descriptions of the included *in vivo* studies and their primary findings; Table S5: General descriptions of the included *in vitro* studies and their primary findings (all preparations are mouth rinse unless otherwise specified); Table S6: Excluded studies and reasons of exclusion; Table S7: Risk of bias assessment of *in vivo* studies; Table S8: Risk of bias assessment of *in vitro* studies. References [19,20,21,22,23,24,25,26,27,28,29,30,31,32,33,34,35,36,37,38,39,40,41,42,43,44,45,46,47,48,49,50,51,54,55,56,57,58,59,60,61,62,63,64,65,66,67,68,69,70,71,72,73,74,75,76,77,78,79,80,81,82,83,84,85,86,87,88,89,90,91,92,93,94,95,96,97,98,99,100,101,102,103,104,105] are cited in the supplementary materials.
