# Supplementary material for: Efficacy of Mouth Rinses and Nasal Spray in the Inactivation of SARS-CoV-2: A Systematic Review and Meta-Analysis of In Vitro and In Vivo Studies
Source: Int J Environ Res Public Health. 2022 Sep 25;19(19):12148. doi: 10.3390/ijerph191912148 (PMC9566636; doi:10.3390/ijerph191912148)
Supplement: Supplementary file 1 [file ijerph-19-12148-s001.zip › ijerph-1893154-supplementary.pdf]

Supplementary materials

Efficacy of mouth rinses and nasal spray in the inactivation of SARS-CoV-2: A systematic review and meta-analysis of *in vitro* and *in vivo* studies

Supplementary Table S1: PRISMA 2020 main checklist

Topic	No.	Item	Location where item is reported
**TITLE**		Efficacy of mouth rinses and nasal spray in the inactivation of SARS-CoV-2: A systematic review and meta-analysis of in vitro and in vivo studies	
**Title**	1	Identify the report as a systematic review.	abstract
**ABSTRACT**			
**Abstract**	2	See the PRISMA 2020 for Abstracts checklist	Supplementary Table 2
**INTRODUCTION**			
**Rationale**	3	Describe the rationale for the review in the context of existing knowledge.	Introduction
**Objectives**	4	Provide an explicit statement of the objective(s) or question(s) the review addresses.	Introduction and 2.2. Focused questions (Materials and Methods)
**METHODS**			
**Eligibility criteria**	5	Specify the inclusion and exclusion criteria for the review and how studies were grouped for the syntheses.	2.3. Eligibility criteria (Materials and Methods)

Topic	No.	Item	Location where item is reported
**Information sources**	6	Specify all databases, registers, websites, organisations, reference lists and other sources searched or consulted to identify studies. Specify the date when each source was last searched or consulted.	2.4. Search strategy and data extraction (Materials and Methods)
**Search strategy**	7	Present the full search strategies for all databases, registers and websites, including any filters and limits used.	Supplementary Table 3
**Selection process**	8	Specify the methods used to decide whether a study met the inclusion criteria of the review, including how many reviewers screened each record and each report retrieved, whether they worked independently, and if applicable, details of automation tools used in the process.	2.4. Search strategy and data extraction (Materials and Methods)
**Data collection process**	9	Specify the methods used to collect data from reports, including how many reviewers collected data from each report, whether they worked independently, any processes for obtaining or confirming data from study investigators, and if applicable, details of automation tools used in the process.	2.4. Search strategy and data extraction (Materials and Methods)
**Data items**	10a	List and define all outcomes for which data were sought. Specify whether all results that were compatible with each outcome domain in each study were sought (e.g. for all measures, time points, analyses), and if not, the methods used to decide which results to collect.	2.4. Search strategy and data extraction (Materials and Methods)
	10b	List and define all other variables for which data were sought (e.g. participant and intervention characteristics, funding sources). Describe any assumptions made about any missing or unclear information.	2.4. Search strategy and data extraction (Materials and Methods)

Topic	No.	Item	Location where item is reported
**Study risk of bias assessment**	11	Specify the methods used to assess risk of bias in the included studies, including details of the tool(s) used, how many reviewers assessed each study and whether they worked independently, and if applicable, details of automation tools used in the process.	2.5. Risk of bias assessment (Materials and Methods)
**Effect measures**	12	Specify for each outcome the effect measure(s) (e.g. risk ratio, mean difference) used in the synthesis or presentation of results.	2.6. Data analysis (Materials and Methods)
**Synthesis methods**	13a	Describe the processes used to decide which studies were eligible for each synthesis (e.g. tabulating the study intervention characteristics and comparing against the planned groups for each synthesis (item 5)).	2.4. Search strategy and data extraction (Materials and Methods)
	13b	Describe any methods required to prepare the data for presentation or synthesis, such as handling of missing summary statistics, or data conversions.	2.4. Search strategy and data extraction (Materials and Methods)
	13c	Describe any methods used to tabulate or visually display results of individual studies and syntheses.	2.4. Search strategy and data extraction (Materials and Methods)
	13d	Describe any methods used to synthesize results and provide a rationale for the choice(s). If meta-analysis was performed, describe the model(s), method(s) to identify the presence and extent of statistical heterogeneity, and software package(s) used.	2.6. Data analysis (Materials and Methods)
	13e	Describe any methods used to explore possible causes of heterogeneity among study results (e.g. subgroup analysis, meta-regression).	2.6. Data analysis (Materials and Methods)
	13f	Describe any sensitivity analyses conducted to assess robustness of the synthesized results.	Materials and Methods

Topic	No.	Item	Location where item is reported
**Reporting bias assessment**	14	Describe any methods used to assess risk of bias due to missing results in a synthesis (arising from reporting biases).	2.5. Risk of bias assessment (Materials and Methods)
**Certainty assessment**	15	Describe any methods used to assess certainty (or confidence) in the body of evidence for an outcome.	Materials and Methods
**RESULTS**			
**Study selection**	16a	Describe the results of the search and selection process, from the number of records identified in the search to the number of studies included in the review, ideally using a flow diagram.	3.1. Results of database searches
	16b	Cite studies that might appear to meet the inclusion criteria, but which were excluded, and explain why they were excluded.	Supplementary Table 6
**Study characteristics**	17	Cite each included study and present its characteristics.	3.2. General characteristics of the included studies AND Supplementary Tables 4 and 5
**Risk of bias in studies**	18	Present assessments of risk of bias for each included study.	Supplementary Tables 7 and 8
**Results of individual studies**	19	For all outcomes, present, for each study: (a) summary statistics for each group (where appropriate) and (b) an effect estimate and its precision (e.g. confidence/credible interval), ideally using structured tables or plots.	Supplementary Tables 4 and 5
**Results of syntheses**	20a	For each synthesis, briefly summarise the characteristics and risk of bias among contributing studies.	3.3. and 3.4. (Results)

Topic	No.	Item	Location where item is reported
	20b	Present results of all statistical syntheses conducted. If meta-analysis was done, present for each the summary estimate and its precision (e.g. confidence/credible interval) and measures of statistical heterogeneity. If comparing groups, describe the direction of the effect.	3.3. and 3.4. (Results)
	20c	Present results of all investigations of possible causes of heterogeneity among study results.	Results
	20d	Present results of all sensitivity analyses conducted to assess the robustness of the synthesized results.	Results
**Reporting biases**	21	Present assessments of risk of bias due to missing results (arising from reporting biases) for each synthesis assessed.	3.2. General characteristics of the included studies (Results)
**Certainty of evidence**	22	Present assessments of certainty (or confidence) in the body of evidence for each outcome assessed.	Results
**DISCUSSION**			
**Discussion**	23a	Provide a general interpretation of the results in the context of other evidence.	Discussion
	23b	Discuss any limitations of the evidence included in the review.	Discussion
	23c	Discuss any limitations of the review processes used.	Discussion
	23d	Discuss implications of the results for practice, policy, and future research.	Discussion
**OTHER INFORMATION**			
**Registration and protocol**	24a	Provide registration information for the review, including register name and registration number, or state that the review was not registered.	2.1. Protocol and registration (Materials and Methods)

Topic	No.	Item	Location where item is reported
	24b	Indicate where the review protocol can be accessed, or state that a protocol was not prepared.	2.1. Protocol and registration (Materials and Methods)
	24c	Describe and explain any amendments to information provided at registration or in the protocol.	N/A
**Support**	25	Describe sources of financial or non-financial support for the review, and the role of the funders or sponsors in the review.	Financial support
**Competing interests**	26	Declare any competing interests of review authors.	Conflicts of Interest
**Availability of data, code and other materials**	27	Report which of the following are publicly available and where they can be found: template data collection forms; data extracted from included studies; data used for all analyses; analytic code; any other materials used in the review.	All materials are publicly available

Supplementary Table S2: PRIMSA 2020 Abstract Checklist

Topic	No.	Item	Reported?
**TITLE**		Efficacy of mouth rinses and nasal spray in the inactivation of SARS-CoV-2: A systematic review and meta-analysis of in vitro and in vivo studies	
**Title**	1	Identify the report as a systematic review.	Yes
**BACKGROUND**			
**Objectives**	2	Provide an explicit statement of the main objective(s) or question(s) the review addresses.	Yes
**METHODS**			
**Eligibility criteria**	3	Specify the inclusion and exclusion criteria for the review.	Yes
**Information sources**	4	Specify the information sources (e.g. databases, registers) used to identify studies and the date when each was last searched.	Yes
**Risk of bias**	5	Specify the methods used to assess risk of bias in the included studies.	Yes
**Synthesis of results**	6	Specify the methods used to present and synthesize results.	Yes
**RESULTS**			
**Included studies**	7	Give the total number of included studies and participants and summarise relevant characteristics of studies.	Yes
**Synthesis of results**	8	Present results for main outcomes, preferably indicating the number of included studies and participants for each. If meta-analysis was done, report the summary estimate and confidence/credible interval. If comparing groups, indicate the direction of the effect (i.e. which group is favoured).	Yes
**DISCUSSION**			

Topic	No.	Item	Reported?
**Limitations of evidence**	9	Provide a brief summary of the limitations of the evidence included in the review (e.g. study risk of bias, inconsistency and imprecision).	Yes
**Interpretation**	10	Provide a general interpretation of the results and important implications.	Yes
**OTHER**			
**Funding**	11	Specify the primary source of funding for the review.	Yes
**Registration**	12	Provide the register name and registration number.	Yes

Supplementary Table S3: MeSH terms used for searching through Pubmed, Scopus, Embase Ovid, and Web of Science databases

Platform	MeSH terms
PubMed	((SARS CoV 2[Title/Abstract] OR SARSCoV2[Title/Abstract] OR SARS-CoV2[Title/Abstract] OR Coronavirus[Title/Abstract] OR Corona[Title/Abstract] OR COVID-19[Title/Abstract] OR COVID 19[Title/Abstract] OR cov2[Title/Abstract] OR Acute Respiratory Syndrome Coronavirus 2[Title/Abstract] OR 2019nCoV[Title/Abstract] OR ncov19[Title/Abstract] OR 2019-novel CoV[Title/Abstract]) AND (Mouth wash[Title/Abstract] OR mouthwash[Title/Abstract] OR oral wash[Title/Abstract] OR mouth rinse[Title/Abstract] OR oral rinse[Title/Abstract] OR spray[Title/Abstract] OR nasal spray[Title/Abstract])) AND (English[Language])
Scopus	(TITLE-ABS-KEY (sars AND cov 2 OR sarscov2 OR sars-cov2 OR coronavirus OR corona OR covid-19 OR covid 19 OR cov2 OR acute AND respiratory AND syndrome AND coronavirus 2 OR 2019ncov OR ncov19 OR 2019-novel AND cov) AND TITLE-ABS-KEY (mouth AND wash OR mouthwash OR oral AND wash OR mouth AND rinse OR oral AND rinse OR spray OR nasal AND spray) AND LANGUAGE (english))
Embase Ovid	((SARS CoV 2 or SARSCoV2 or SARS-CoV2 or Coronavirus or Corona or COVID-19 or COVID 19 or cov2 or Acute Respiratory Syndrome Coronavirus 2 or 2019nCoV or ncov19 or 2019-novel CoV) and (Mouth wash or mouthwash or oral wash or mouth rinse or oral rinse or spray or nasal spray)).ab.
Web of Science	(((AB=(SARS CoV 2 OR SARSCoV2 OR SARS-CoV2 OR Coronavirus OR Corona OR COVID-19 OR COVID 19 OR cov2 OR Acute Respiratory Syndrome Coronavirus 2 OR 2019nCoV OR ncov19 OR 2019-novel CoV)) AND AB=(Mouth wash OR mouthwash OR oral wash OR mouth rinse OR oral rinse OR spray OR nasal spray))) AND DOP=(2019-12-01/2022-04-15)

Supplementary Table S4: General descriptions of the included *in vivo* studies and their primary findings.

Study (year)	Country	Active ingredients / concentration	Route	N	values	Main results
**Carrouel *et al*. 2021**[1]	France	CDCM	Mouth rinse	76	log_{10} copies/ mL	Using CDCM significantly reduced the salivary viral load for at least 4 hours. For long term effects after washing with CDCM once a day for 7 days, CDCM reduced the salivary viral load but not significantly.
**Costa *et al*. 2021**[2]	Brazil	CHX 0.12%	Mouth rinse	50	Ct	CHX significantly reduced the viral load for up to 60 minutes in comparison to a control group
**Eduardo *et al*. 2021**[3]	Brazil	CHX	Mouth rinse	8	Ct	Rinsing with CHX or with CPC+Zn significantly reduced the viral load for up to 60 minutes, while HP mouth rinse was associated with a significant reduction for 30 minutes.
		CHX + HP	Mouth rinse	11		
		HP	Mouth rinse	6		
		CPC+ Zn	Mouth rinse	7		
**Elzein *et al*. 2021**[4]	Lebanon	CHX 0.2%	Mouth rinse	27	Ct	Both CHX and PVP-I showed a significant reduction in the viral load, $p < 0.0001$. However, the mean difference of Ct values in the paired samples was higher for CHX in comparison to PVP-I, 5.69 and 4.45, respectively.
		PVP-I 1.0%	Mouth rinse	25		
**Gottsauner *et al*. 2020**[5]	Germany	HP 1.0%	Mouth rinse	10	Copies/ mL	Using HP 1.0% reduced the mean value of viral load from 1.8 x 10^3 to 1.5 x 10^3 copies/ mL, however, this reduction was not significant.
**Guimarães *et al*. 2021**[6]	Brazil	HP 1.5%	Mouth rinse	12	Copies/ µL	None of the preparations showed a significant difference in the viral load in comparison to a placebo group. However, HP 1.5% was associated with a significant reduction in the viral load after 30 minutes in comparison to the basal measurements.
		CHX 0.12%	Mouth rinse	12		
		NaClO 0.1%	Mouth rinse	12		
		CHX (0.12%) + HP (1.5%)	Mouth rinse	12		
**Lamas *et al*. 2020**[7]	Spain	PVP-I 1.0%	Mouth rinse	4	log_{10} copies/ mL	Using PVP-I 1.0% was associated with a significant reduction in the salivary viral load that remained for at least 3 hours.
	Singapore	PVP-I 0.5%	Mouth rinse	4	Ct	

**Seneviratne *et al.* 2021**[8]		CHX 0.2%	Mouth rinse	6		No statistical differences were found in the Ct values for all preparations. However, both PVP-I 0.5% and CPC showed significant differences in the viral load at certain time points in comparison to water.
		CPC	Mouth rinse	4		
**Schürmann *et al.* 2021**[9]	Germany	Linola sept,	Mouth rinse	29	Ct	A significant reduction in viral load was reported. The difference in the mean value of Ct was 3.1, which indicated a 90% reduction in the viral load in the pharynx.
**Yoon et al. 2020**[10]	Korea	CHX 0.12%	Mouth rinse	2	log_{10} copies/ mL	Viral dynamics in various body fluids were checked. The viral load was the highest in the nasopharynx and saliva in comparison to the oropharynx, sputum, and urine. The salivary viral load decreased for 2 hours after CHX rinsing but increased again at 2-4 hours.
**Zarabanda *et al.* 2021**[11]	USA	PVP-I 0.5%	Nasal spray	11	Ct	A significant reduction in viral load was reported. The mean difference of Ct values was higher for PVP-I 0.5% in comparison to PVP-I 2.0%, - 0.349 and – 1.059, respectively.
		PVP-I 2.0%	Nasal spray	11		
N; the number of subjects, CDCM; b-cyclodextrinecitrox mouthwash, Ct; cycle threshold in qPCR assays, CHX; chlorhexidine, HP; hydrogen peroxide, CPC; cetylpyridinium chloride, Zn; Zinc, PVP-I; povidone-iodine, NaClO; sodium hypochlorite, Linola sept; Linola sept, Dr. August Wolff GmbH (commercial name).						

Supplementary Table S5: General descriptions of the included *in vitro* studies and their primary findings (all preparations are mouth rinse unless otherwise specified)

Study	Active ingredients / concentrations	Methods	Major findings
**Anderson *et al*. 2020**[12]	(PVP-I 0.5%), (PVP-I 1.0%)	SARS-CoV-2 (hCoV19/ Singapore/2/2020) was cultivated in Vero-E6 cells. The virus was exposed to the test products at 21°C for 30 seconds. Experiments were carried out in triplicates.	All test products showed a ≥ 4 LRV of the virus titres, which is corresponding to 99.99%.
**Anderson *et al*. 2022**[13]	(CPC 0.07% + herbs extracts), (CPC 0.07%), (CHX 0.2%)	SARS-CoV-2 (USA-WA1/2020) in Vero E6 cells. The virus was exposed to the test products for 30 seconds at room temperature. Tests were performed in triplicates.	CPC preparations were associated with ≥ 4 LRV of the virus titres, while CHX showed < 2.0 LRV.
**Bidra *et al*. (1) 2020**[14]	(PVP-1 1.5%), (PVP-1 0.75%), (PVP-1 0.5%), (EtOH 70%)	SARS-CoV-2 (USA-WA1/2020) was propagated in Vero 76 cells. The virus was exposed to the test products at room temperature for 15 and 30 seconds. Experiments were carried out in triplicates.	The LRVs for all PVP-I preparations were 3.0 at 15 seconds and 3.33 at 30 seconds. EtOH 70% was associated with an LRV of 2.17 at 15 seconds and 3.33 at 30 seconds.
**Bidra (2) *et al*. 2020**[15]	(PVP-I 0.5%), (PVP-I 1.25%), (PVP-I 1.5%), (HP 1.5%), (HP 3.0%)	SARS-CoV-2 (USA-WA1/2020) in Vero 76 cells. The virus was exposed to the test products at room temperature for 15 and 30 seconds. Experiments were carried out in triplicates.	PVP-I preparations were associated with >4.33 LRVs at 15 seconds and >3.63 LRV at 30 seconds. The LRVs for HP 1.5% at 15 and 30 seconds were 1.33 and 1.0, respectively. The LRVs for HP 3.0% at 15 and 30 seconds were 1.0 and 1.8, respectively.
**Davies *et al*. 2021**[16]	(CHX 0.2%), (CHX 0.2% + EtOH), (Listerine Advanced®: dipotassium oxalate 1.4%), (Listerine Total Care®: NaF, ZnF_2, menthol, thymol), (HClO 0.01-0.02%),	SARS-CoV-2 England 2 strain propagated in Vero E6 cells. The virus was exposed to the test products at room temperature for 60 seconds. All products were tested in triplicates.	The highest LRV (≥ 5.5) was associated with HClO and followed by PVP-I and Listerine Total Care® (≥ 4.1). The LRV of Listerine Advanced® was ≥ 3.5. Other products associated with LRVs ≤ 0.5.

	(HP 1.5%),<br>(PVP-I 0.58%)		
**Frank *et al*. 2020a[17]**	(PVP-I 2.5%),<br>(PVP-I 1.25%),<br>(PVP-I 0.5%)<br>(All were nasal sprays)	SARS-CoV-2 (USA-WA1/2020) strain was grown in Vero 76 cells. The test solutions and the virus were incubated at room temperature for 15 and 30 seconds. Each concentration was tested in triplicate.	The LRVs for all test products were 3.0 when incubated for 15 seconds and 3.33 when incubated for 30 seconds.
**Gudmundsdottir *et al* 2020[18]**	(Mouth spray ColdZyme®: glycerol, trypsin, EtOH <1%)	SARS-CoV-2 (USA-WA1/2020) strain was grown in Vero 76 cells. Tests were done in duplicate.	The tested product was associated with LRV = 1.76, which is equivalent to the inactivation of 98.3% of virus titres.
**Hassandarvish *et al*. 2020[19]**	(PVP-I 0.5%),<br>(PVP-I 1.0%)	SARS-CoV-2 (SARS-COV-2/ MY/ UM/6-3) cultivated in Vero E6 cells. Virus inactivation tests were performed under clean and dirty conditions for 15, 30, and 60 seconds. Dirty conditions were stimulated by adding human erythrocytes to simulate organic soiling.	PVP-I 1.0% was associated with LRVs > 5 for all time frames. The LRVs were > 4 for PVP-I 0.5% at 15 seconds and > 5 at 30 and 60 seconds. LRVs were not changed between the clean and dirty conditions.
**Kariwa *et al*. 2020[20]**	(PVP-I 0.23%),<br>(PVP-I 0.35%),<br>(PVP-I 0.45%)	SARS-CoV-2 (WK-521 strain) was propagated in Vero E6 cells. The virus was exposed to the test products for 30 and 60 seconds.	All preparations were associated with LRVs > 3.1. This is corresponding to inactivation of > 99.92.
**Koch-Heier *et al*. 2021[21]**	(HP 1.5% + CPC 0.05%),<br>(CHX 0.1% + CPC 0.05%),<br>(CHX 0.1% + CPC 0.05%),<br>(CHX 0.1%),<br>(HP 1.5%),<br>(CPC 0.05%)	SARS-CoV-2; Isolate “FI-100” was propagated in Vero E6 cells. The virus was exposed to test products for 30 seconds at 37°C. Each product was tested in duplicates.	The highest LRVs were reported for HP 1.5% + CPC 0.05% and CHX 0.1% + CPC 0.05% (LRV ≥1.9). CHX 0.1% + CPC 0.05% and CPC 0.05% were associated with LRVs equal 1.2 and 0.7, respectively. Other test products did not show reduction in the virus titre.
**Komine *et al*. 2021[22]**	(CHX 0.06% + CPC 0.05%),<br>(CHX 0.12% + CPC 0.05%),<br>(CPC 0.075% CHX 0.12%),	SARS-CoV-2 (JPN/TY/WK-521 strain) was propagated in Vero E6 cells. The virus was incubated with the test products at 25°C for 20, 30, and 180 seconds. All experiments were performed in triplicates.	All the products containing<br>CPC inactivated the virus between 3.3 to > 4.4 LRV. The LRV associated with Del hydrochloride was > 5.4. A test product with only CHX did not show sufficient inactivation capacity against the virus, LRV = 0.2.

	(Del hydrochloride, CPC 0.05%)		
**Kontos 2021**[23]	(essential iodine drops 50%), (essential iodine drops 66%), (essential iodine drops 75%)	SARS-CoV-2 (USA-WA1/2020) in Vero 76 cells. Test products were tested in triplicates at room temperature for 60 and 90 seconds.	At 60 seconds, the concentrations of 66% and 75% were associated with LRVs equal to 1.7 and 2.0, respectively. The LRV of the 50% concentration was 2.0 for 90 seconds.
**Liang *et al*. 2020**[24]	(PVP-I 0.9%), (PVP-I 0.5%), (PVP-I 0.28%), (PVP-I 0.09%), (PVP-I 0.54%), (PVP-I 0.3%), (PVP-I 0.17%), (PVP-I 0.05%)	SARS-CoV-2 (USA-WA1/2020) was propagated in Vero 76 cells. The virus was incubated with the tested concentrations at 37°C for 30 seconds, 2, and 10 minutes. All experiments were performed in triplicates.	The concentrations ≥ 0.3% were associated with LRVs ≥ 3.1 for 30 seconds. Other concentrations associated with LRVs ranged between 1.2 to 2.2 for 30 seconds. Increasing the exposure time did not increase the LRVs.
**Meister *et al*. 2022**[25]	(0.4mg/ml $NaClO_2$), ($NaClO_2$ 0.9%, panthenol), (dexpanthenol 50 mg/mL), ($NaClO_2$<0.08%, Xylometazolin hydrochloride 0.1%), (disodium succinate)	SARS-CoV-2 (hCoV-19/ Germany/BY-Bochum-1/2020) cultivated in Vero E6 cells. The virus was inactivated by the test products for 30 seconds. All experiments were performed in triplicates.	Only Sodium hypochlorite ($NaClO_2$ <0.05%) lowered the viral titres by 2.21. The LRVs for all other products ranged from 0.18 to 0.53.
**Pelletier *et al*. 2021**[26]	(nasal spray PVP-I 2.5%), (nasal spray PVP-I 1.25%),	SARS-CoV-2 (USA-WA1/2020) was grown in Vero 76 cells. The virus was incubated with the test products for 60 seconds at room temperature. All products were tested in triplicates.	The LRVs for all tested products were 4.63 at 60 seconds.

	(nasal spray PVP-I 0.5%), (PVP-I 1.5%), (PVP-I 0.75%), (PVP-I 0.5%)		
**Pyrć *et al*. 2021[27]**	(GCPQ molecular weight=10kDa), (GCPQ molecular weight=30kDa), (GCPQ molecular weight=15kDa), (GCPQ molecular weight=60kDa)	SARS-CoV-2 (isolate 026 V-03883) was propagated in Vero E6 cells and A549 cells. All tests were carried out in triplicates.	The highest LRVs were associated with molecular weights 10kDa and 15kDa, 3.87 and 1.79, respectively. The LRVs of the other preparations were ≤ 0.24.
**Santos *et al*. 2021[28]**	(Mouth rinse 0.1% APD), (Dental gel 1.0% APD)	Samples of SARS-CoV-2 were collected from oropharyngeal samples and propagated in Vero CCL-81 cells. The virus was inactivated with the tested products for 30. 60, and 300 seconds.	The mouth rinse was associated with LRV = 4.5, corresponding to 99.99% of viral inactivation. The dental gel showed lower efficiency with LRV = equals 1.5, corresponding to 90% of viral inactivation.
**Shet *et al*. 2022[29]**	(PVP-I 0.5%)	SARS-CoV-2 (USA-WA1/2020) in Vero 76 cells. The virus was inactivated by the test product at room temperature for 15, 30, 60, and 300 seconds. The experiment was repeated in triplicates.	The LRV was > 4 at 30 and 300 seconds. At 15 and 60 seconds, the LRVs were 2.8 and 3.67, respectively.
**Shewale *et al*. 2021[30]**	All products are commercially from CloSYS: Toothpaste, (NaF 0.24%, Sensitive Mouthwash ClO_2, Na_3PO_4, citric acid), (Ultra-Sensitive Mouthwash	SARS-CoV-2 (USA-WA1/2020) in Vero E6 cells. The virus was inactivated by the test products 30, 60 and/or 120 seconds at room temperature.	The toothpaste was associated with LRV = 2.26 for 30, 60, and 120 seconds, which is equivalent to the inactivation of 99.4% of the virus. The sensitive mouthwash showed LRV equals 1.81 and 1.71 at 30 and 60 seconds, respectively, which is corresponding to the inactivation of≥ 98% of the virus. The LRVs for the ultra-sensitive mouthwash were 1.96 at 30 seconds and 1.39 at 60 seconds, which is corresponding to the inactivation of 98.4% and 96.3% of the virus, respectively. Finally, The oral spray was associated with LRV equals 2.98 at 30 seconds and 2.67 at 60 seconds, which is

	ClO_2, Na_3PO_4, citric acid), (Oral Spray ClO_2, Na_3PO_4, citric acid)		corresponding to the inactivation of 99.9% and 99.7% of the virus, respectively.
**Steinhauer *et al*. 2021**[31]	(CHX 0.1%), (CHX 0.2%)	SARS-CoV-2 was inactivated by the tested products for 1 to 10 minutes. The experiments were repeated in duplicates.	The concertation of 0.1% was associated with LRV <1 at 10 minutes while the 0.2% was associated with LRV <1 at 1 and 5 minutes.
**Teagle *et al.* 2022**[32]	(Molecular iodine)	SARS-CoV-2 (USA-WA1/2020) was propagated in Vero E6 cells. The virus was inactivated by the tested product with/without the presence of saliva for 30 and 60 seconds.	The LRV was 4.75 at 30 seconds and ≥5.25 at 60 seconds with the presence of saliva. Without saliva, the LRV was ≥5.75 at 30 and 60 seconds. The reported LRVs are equivalent to inactivation of >99.99% of virus.
**Tiong *et al*. 2021**[33]	(CHX 0.12%), (CPC 0.075%+ NaF 0.05%), (thymol 0.05%), (Hexetidine 0.1%+EtOH 9%, NaCl 2% 0.4M)	SARS-CoV-2 was isolated from a nasopharyngeal/oropharyngeal swap sample (SARS-COV-2/MY/UM/6-3; TIDREC) of a positive individual and propagated in Vero E6 cells. The virus was inactivated by the test products under 2 conditions, clean and dirty (by adding human erythrocytes) for a period of 30 and 60 seconds.	CHX preparation was associated with LRV = 4 for 30 and 60 seconds under dirty and clean conditions. CPC and hexetidine preparations showed LRVs equal to 5 for 30 and 60 seconds and under all conditions. Under the clean condition, thymol preparation was associated with LRV = 0.5 at 30 seconds and LRV = 0.75 at 60 seconds. For the dirty condition, the LRV for the thymol preparation was 0.5 at 30 and 60 seconds.
PVP-I; povidone-iodine, CPC; cetylpyridinium chloride, CHX; chlorhexidine, EtOH; ethanol, HP; hydrogen peroxide, SDS; sodium dodecyl sulfate, NaF; sodium fluoride, ZnF_2; zinc difluoride, HClO; hypochlorous acid, $NaClO_2$; sodium chlorite, GCPQ; a polymer name (N-palmitoyl-N-monomethyl-N,N-dimethyl-N,N,N-trimethyl-6-O-glycolchitosan), ADP; anionic phthalocyanine derivate, ClO_2; chlorine dioxide, Na_3PO_4; trisodium phosphate, NaCl; sodium chloride.			

Supplementary Table S6: Excluded studies and reasons of exclusion

Study	Reason of exclusion
**Abdelalim et al. (2021)** [34]<br>**Chaudhary et al. (2021)**[35]<br>**Burgos-Ramos et al. (2022)**[36]<br>**Orcina et al. (2021)**[37]<br>**Di Domênico et al. (2021)**[38]<br>**Figueroa et al. (2021)**[39]<br>**Guenezan et al. (2021)**[40]<br>**Huang and Huang (2021)**[41]<br>**Kasiri et al. (2021)**[42]<br>**Arefin et al. (2021)**[43]<br>**Khan et al. (2020)**[44]<br>**Saud et al. (2022)**[45]<br>**Avhad et al. (2020)**[46]<br>**Almanza-Reyes et al. (2021)**[47]<br>**Aref et al. (2021)**[48]	Subject-level reports of SARS-CoV-2 viral load was not mentioned
**Laferl et al. (2022)**[49]<br>**Michel et al. (2021)**[50]<br>**Mora-Aguilera (2022)**[51]	Papers for diagnostic rather than therapeutic purposes
**Paull et al. (2021)**[52]<br>**Errecalde et al. (2021)**[53]	*In vivo* animal study
**Jain et al. (2021)**[54]	The number of replicates is not mentioned
**Bañó-Polo et al. (2022)**[55]<br>**Bansal et al. (2021)**[56]<br>**Bentley et al. (2021)**[57]<br>**Bovard et al. (2022)**[58]<br>**Haridas et al. (2021)**[59]<br>**Moakes et al. (2021)**[60]<br>**Morokutti-Kurz et al (2021)**[61]	LRV between control and experimental groups is not mentioned

**Muñoz-Basagoiti et al (2021)[62]**<br>**Paolacci et al. (2021)[63]**<br>**Rodriguez et al. (2021)[64]**<br>**Sharad and Kapur (2021)[65]**<br>**Tateyama-Makino et al. (2021)[66]**<br>**Yadalam et al. (2021)[67]**<br>**Casanovas et al. (2021)[68]**<br>**Robinson et al. (2021)[69]**	
**Westover et al. (2020)[70]**<br>**Cannon et al. (2020)[71]**<br>**Mohamed et al. (2020)[72]**<br>**Statkute et al. (2020)[73]**<br>**Xu et al. (2021)[74]**	High Contact time (incompatible with EN 14476:2013+A2:2019), not peer reviewed
**Buonavoglia et al. (2021)[75]**<br>**Green et al. (2020)[76]**<br>**Shet et al. (2021)[77]**	Used of SARS-CoV-2 surrogates
**Balouch et al. (2021)[78]**<br>**Frank et al. (2020b)[79]**<br>**Vergara-Buenaventura and Castro-Ruiz (2020)[80]**<br>**Telles-Araujo et al. (2020)[81]**<br>**Gerlach et al. (2020)[82]**<br>**Carrouel et al. (2020)[83]**<br>**Peng et al. (2020)[84]**<br>**Pattanshetty et al. (2021)[85]**	Not experimental, expert opinions, or commentaries

Supplementary Table S7: Risk of bias assessment of *in vivo* studies

Study	ToxRTool items, *in vivo* (0 = no, 1 = yes)															Total
	1	2	3	4	5	6	7	8	9	10	11	12	13	14	15	
**Carrouel et al. 2021[1]**	1	1	1	0	1	1	1	1	1	1	1	1	1	1	1	**14**
**Costa et al. 2021[2]**	1	1	0	0	1	1	1	1	1	1	1	1	1	1	0	**13**
**Eduardo et al. 2021[3]**	1	1	1	0	1	1	1	1	1	1	1	1	1	1	0	**13**
**Elzein et al. 2021[4]**	1	1	0	0	1	1	1	1	1	1	1	1	1	1	0	**12**
**Gottsauner et al. 2020[5]**	1	1	0	0	1	1	1	1	1	1	1	1	1	1	0	**12**
**Guimarães et al. 2021[6]**	1	1	0	0	1	1	1	1	1	1	1	1	1	1	0	**12**
**Lamas et al. 2020[7]**	1	1	0	0	1	1	1	1	1	1	1	1	1	1	0	**12**
**Seneviratne et al. 2021[8]**	1	1	1	0	1	1	1	1	1	1	1	1	1	1	0	**13**
**Schürmann et al. 2021[9]**	1	1	1	0	1	1	1	1	1	1	1	1	1	1	1	**14**
**Yoon et al. 2020[10]**	1	1	0	0	1	1	1	1	1	1	1	1	1	1	1	**13**
**Zarabanda et al. 2021[11]**	1	1	0	0	1	1	1	1	1	1	1	1	1	1	0	**13**

Legend for Supplementary Table S7:

The Risk of Bias Assessment for *in vivo* studies was done using the Toxicological data reliability assessment tool (ToxRTool, *in vivo* part). The following are the questions that were used for this purpose:

1. Was the test substance identified?
2. Was the purity/concentration of the substance given?
3. Was the information on the source/origin of the substance given?
4. Was the virus strain (a strain of SARS-CoV-2) given?
5. Was the administration route given?

6. Were frequency and duration of exposure as well as time-points of observations explained?
7. Were negative (where required) and positive controls (where required) included?
8. Was the number of subjects per group given?
9. Were the study endpoint(s) and their method(s) of determination clearly described?
10. Were sufficient details of the administration scheme given to judge the study?
11. Was the description of the study results for all endpoints investigated transparent and complete?
12. Were the statistical methods applied for data analysis given and applied in a transparent manner?
13. Was the study design chosen appropriately for obtaining the substance-specific data aimed at?
14. Were the quantitative study results reliable?
15. Were used primers, probes, and standard errors, explained clearly?

Supplementary Table S8: Risk of bias assessment of *in vitro* studies

Study	ToxRTool items, *in vitro* (0 = no, 1 = yes)																	Total
	**1**	**2**	**3**	**4**	**5**	**6**	**7**	**8**	**9**	**10**	**11**	**12**	**13**	**14**	**15**	**16**	**17**	
**Anderson *et al.* 2020**[12]	1	1	1	1	1	1	1	1	1	1	1	1	1	1	1	1	1	**17**
**Anderson *et al.* 2022**[13]	1	1	0	1	1	1	1	1	1	1	1	1	1	1	1	1	1	**16**
**Bidra *et al*. (1) 2020**[14]	1	1	1	1	1	0	1	1	1	1	1	1	1	1	1	1	1	**16**
**Bidra (2*) et al*. 2020**[15]	1	1	1	1	1	0	1	1	1	1	1	1	1	1	1	1	1	**16**
**Davies *et al.* 2021**[16]	1	1	1	1	1	0	1	1	1	1	0	1	1	1	1	1	1	**15**
**Frank et al. 2020a**[17]	1	1	1	1	1	0	1	1	1	1	1	1	1	1	1	1	1	**16**
**Gudmundsdottir *et al.* 2020**[18]	1	1	1	1	1	1	1	1	1	1	0	1	1	1	1	1	1	**16**
**Hassandarvish *et al.* 2020**[19]	1	1	0	1	1	1	1	1	1	1	0	1	1	1	1	1	1	**15**
**Kariwa *et al*. 2020**[20]	1	1	0	1	1	0	1	1	1	1	0	1	1	1	1	1	1	**14**
**Koch-Heier *et al.* 2021**[21]	1	1	1	1	1	1	1	1	1	1	0	1	1	1	1	1	1	**16**
**Komine *et al*. 2021**[22]	1	1	1	1	1	1	1	1	1	1	1	1	1	1	1	1	1	**17**
**Kontos 2021**[23]	1	1	1	1	1	1	1	1	1	1	1	1	1	1	1	1	1	**17**
**Liang *et al*. 2020**[24]	1	1	0	1	1	1	1	1	1	1	1	1	1	1	1	1	1	**16**
**Meister *et al*. 2022**[25]	1	1	0	1	1	0	1	1	1	1	0	1	1	1	1	1	1	**15**
**Pelletier *et al*. 2021**[26]	1	1	1	1	1	1	1	1	1	1	0	1	1	1	1	1	1	**16**
**Pyrć *et al*. 2021**[27]	1	1	1	1	1	0	1	1	1	1	0	1	1	1	1	1	1	**15**
**Santos *et al*. 2021**[28]	1	1	1	1	1	1	1	1	1	1	1	1	1	1	1	1	1	**17**
**Shet *et al*. 2022**[29]	1	1	0	1	1	1	1	1	1	1	1	1	1	1	1	1	1	**16**
**Shewale *et al*. 2021**[30]	1	1	1	1	1	1	1	1	1	1	0	1	1	1	1	1	1	**16**
**Steinhauer *et al.* 2021**[31]	1	1	1	1	1	0	0	1	1	1	0	1	1	1	1	1	1	**15**
**Teagle *et al.* 2022**[32]	1	1	0	1	1	1	1	1	1	1	1	1	1	1	1	1	1	**16**
**Tiong *et al*. 2021**[33]	1	1	1	1	1	0	1	1	1	1	0	1	1	1	1	1	1	**15**

Legend for Supplementary Table S8:

The Risk of Bias Assessment for *in vitro* studies was done using the Toxicological data reliability assessment tool (ToxRTool, *in vitro* part). The following are the questions that were used for this purpose:

1. Was the test substance identified?
2. Was the purity/concentration of the substance given?
3. Was the information on the source/origin of the substance given?
4. Was the test system described (type of cells or tissue used: primary cells, cell lines)?
5. Was the strain of SARS-CoV-2 given?
6. Was information given on the source/origin of the test system (laboratory/scientist/company providing cell lines)?
7. Was necessary information on test system properties, and conditions of cultivation and maintenance given?
8. Was the method of administration given?
9. Were frequency and duration of exposure as well as time-points of observations explained?
10. Were negative controls included?
11. Were positive controls included?
12. Was the number of replicates (or complete repetitions of the experiment) given?
13. Were the study endpoint(s) and their method(s) of determination clearly described?
14. Was the description of the study results for all endpoints investigated transparent and complete?
15. Were doses administered or concentrations in application media given?
16. Was the study design chosen appropriately for obtaining the substance-specific data aimed at?
17. Are the quantitative study results reliable?

Supplementary Figure S1: Funnel plot of the standard error by difference in means for *in vitro* studies

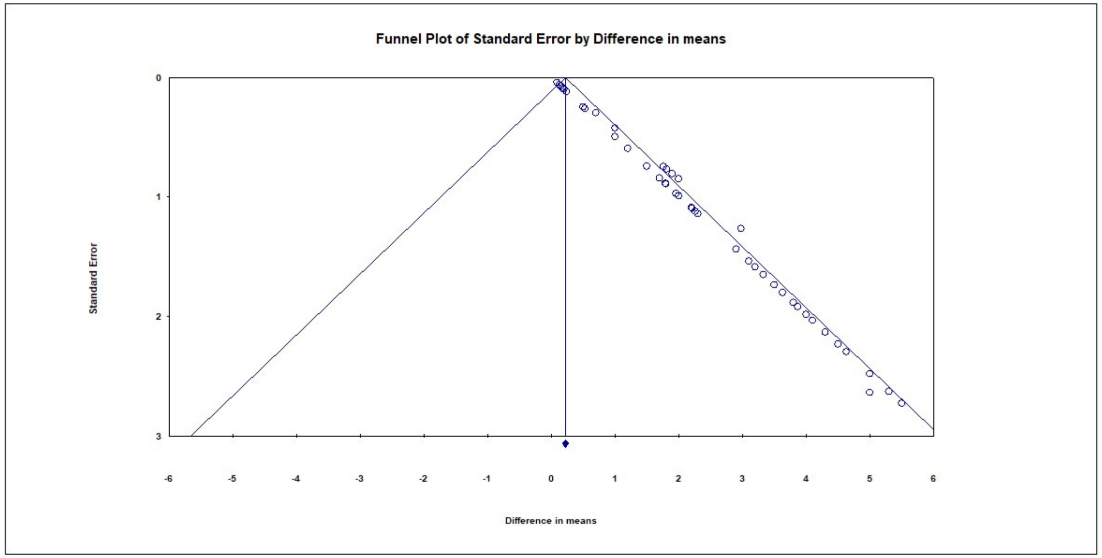

Model		Effect size and 95% confidence interval					Test of null (2-Tail)		Heterogeneity				Tau-squared			
Model	Number Studies	Point estimate	Standard error	Variance	Lower limit	Upper limit	Z-value	P-value	Q-value	df (Q)	P-value	I-squared	Tau Squared	Standard Error	Variance	Tau
Fixed	79	0.220	0.025	0.001	0.171	0.268	8.885	0.000	253.179	78	0.000	69.192	0.127	0.072	0.005	0.356
Random effects	79	0.886	0.089	0.008	0.712	1.060	9.982	0.000								

References

1. Carrouel F, Valette M, Gadea E, et al. Use of an antiviral mouthwash as a barrier measure in the SARS-CoV-2 transmission in adults with asymptomatic to mild COVID-19: a multicentre, randomized, double-blind controlled trial. *Clin Microbiol Infect.* 2021;27(10):1494-1501.
2. Costa DD, Brites C, Vaz SN, de Santana DS, Dos Santos JN, Cury PR. Chlorhexidine mouthwash reduces the salivary viral load of SARS-CoV-2: A randomized clinical trial. *Oral Dis.* 2021.
3. Eduardo FP, Correa L, Heller D, et al. Salivary SARS-CoV-2 load reduction with mouthwash use: A randomized pilot clinical trial. *Heliyon.* 2021;7(6):e07346.
4. Elzein R, Abdel-Sater F, Fakhreddine S, et al. In vivo evaluation of the virucidal efficacy of chlorhexidine and povidone-iodine mouthwashes against salivary SARS-CoV-2. A randomized-controlled clinical trial. *J Evid Based Dent Pract.* 2021;21(3):101584.
5. Gottsauner MJ, Michaelides I, Schmidt B, et al. A prospective clinical pilot study on the effects of a hydrogen peroxide mouthrinse on the intraoral viral load of SARS-CoV-2. *Clin Oral Investig.* 2020;24(10):3707-3713.
6. Guimaraes TC, Marques BBF, Castro MV, et al. Reducing the viral load of SARS-CoV-2 in the saliva of patients with COVID-19. *Oral Dis.* 2021.
7. Martinez Lamas L, Diz Dios P, Perez Rodriguez MT, et al. Is povidone iodine mouthwash effective against SARS-CoV-2? First in vivo tests. *Oral Dis.* 2020;28 Suppl 1:908-911.
8. Seneviratne CJ, Balan P, Ko KKK, et al. Efficacy of commercial mouth-rinses on SARS-CoV-2 viral load in saliva: randomized control trial in Singapore. *Infection.* 2021;49(2):305-311.
9. Schurmann M, Aljubeh M, Tiemann C, Sudhoff H. Mouthrinses against SARS-CoV-2: anti-inflammatory effectivity and a clinical pilot study. *Eur Arch Otorhinolaryngol.* 2021;278(12):5059-5067.
10. Yoon JG, Yoon J, Song JY, et al. Clinical Significance of a High SARS-CoV-2 Viral Load in the Saliva. *J Korean Med Sci.* 2020;35(20):e195.
11. Zarabanda D, Vukkadala N, Phillips KM, et al. The Effect of Povidone-Iodine Nasal Spray on Nasopharyngeal SARS-CoV-2 Viral Load: A Randomized Control Trial. *Laryngoscope.* 2021.
12. Anderson DE, Sivalingam V, Kang AEZ, et al. Povidone-Iodine Demonstrates Rapid In Vitro Virucidal Activity Against SARS-CoV-2, The Virus Causing COVID-19 Disease. *Infect Dis Ther.* 2020;9(3):669-675.
13. Anderson ER, Patterson EI, Richards S, et al. CPC-containing oral rinses inactivate SARS-CoV-2 variants and are active in the presence of human saliva. *J Med Microbiol.* 2022;71(2).
14. Bidra AS, Pelletier JS, Westover JB, Frank S, Brown SM, Tessema B. Rapid In-Vitro Inactivation of Severe Acute Respiratory Syndrome Coronavirus 2 (SARS-CoV-2) Using Povidone-Iodine Oral Antiseptic Rinse. *J Prosthodont.* 2020;29(6):529-533.
15. Bidra AS, Pelletier JS, Westover JB, Frank S, Brown SM, Tessema B. Comparison of In Vitro Inactivation of SARS CoV-2 with Hydrogen Peroxide and Povidone-Iodine Oral Antiseptic Rinses. *J Prosthodont.* 2020;29(7):599-603.
16. Davies K, Buczkowski H, Welch SR, et al. Effective in vitro inactivation of SARS-CoV-2 by commercially available mouthwashes. *J Gen Virol.* 2021;102(4).

17. Frank S, Brown SM, Capriotti JA, Westover JB, Pelletier JS, Tessema B. In Vitro Efficacy of a Povidone-Iodine Nasal Antiseptic for Rapid Inactivation of SARS-CoV-2. *JAMA Otolaryngol Head Neck Surg.* 2020;146(11):1054-1058.
18. Gudmundsdottir A, Scheving R, Lindberg F, Stefansson B. Inactivation of SARS-CoV-2 and HCoV-229E in vitro by ColdZyme(R) a medical device mouth spray against the common cold. *J Med Virol.* 2021;93(3):1792-1795.
19. Hassandarvish P, Tiong V, Mohamed NA, et al. In vitro virucidal activity of povidone iodine gargle and mouthwash against SARS-CoV-2: implications for dental practice. *Br Dent J.* 2020.
20. Kariwa HS, H.; Kobayashi, S. Inactivation of sars-cov-2 by povidone-iodine products: Implications for effective mouth rinsing and gargling. *apanese Journal of Veterinary Research.* 2021;69(3):183-187.
21. Koch-Heier J, Hoffmann H, Schindler M, Lussi A, Planz O. Inactivation of SARS-CoV-2 through Treatment with the Mouth Rinsing Solutions ViruProX((R)) and BacterX((R)) Pro. *Microorganisms.* 2021;9(3).
22. Komine A, Yamaguchi E, Okamoto N, Yamamoto K. Virucidal activity of oral care products against SARS-CoV-2 in vitro. *J Oral Maxillofac Surg Med Pathol.* 2021;33(4):475-477.
23. Kontos Z. Efficacy of "Essential Iodine Drops" against Severe Acute Respiratory Syndrome-Coronavirus 2 (SARS-CoV-2). *PLoS One.* 2021;16(7):e0254341.
24. Liang B, Yuan X, Wei G, et al. In-Vivo Toxicity Studies and In-Vitro Inactivation of SARS-CoV-2 by Povidone-iodine In-situ Gel Forming Formulations. *bioRxiv.* 2020.
25. Meister TL, Todt D, Bruggemann Y, et al. Virucidal activity of nasal sprays against severe acute respiratory syndrome coronavirus-2. *J Hosp Infect.* 2022;120:9-13.
26. Pelletier JS, Tessema B, Frank S, Westover JB, Brown SM, Capriotti JA. Efficacy of Povidone-Iodine Nasal and Oral Antiseptic Preparations Against Severe Acute Respiratory Syndrome-Coronavirus 2 (SARS-CoV-2). *Ear Nose Throat J.* 2021;100(2_suppl):192S-196S.
27. Pyrc K, Milewska A, Duran EB, et al. SARS-CoV-2 inhibition using a mucoadhesive, amphiphilic chitosan that may serve as an anti-viral nasal spray. *Sci Rep.* 2021;11(1):20012.
28. Santos C, da Fonseca Orcina B, Brito Reia VC, et al. Virucidal Activity of the Antiseptic Mouthwash and Dental Gel Containing Anionic Phthalocyanine Derivative: In vitro Study. *Clin Cosmet Investig Dent.* 2021;13:269-274.
29. Shet M, Westover J, Hong R, Igo D, Cataldo M, Bhaskar S. In vitro inactivation of SARS-CoV-2 using a povidone-iodine oral rinse. *BMC Oral Health.* 2022;22(1):47.
30. Shewale JG, Gelhaus HC, Ratcliff JL, Hernandez-Kapila YL. In vitro antiviral activity of stabilized chlorine dioxide containing oral care products. *Oral Dis.* 2021.
31. Steinhauer K, Meister TL, Todt D, et al. Comparison of the in-vitro efficacy of different mouthwash solutions targeting SARS-CoV-2 based on the European Standard EN 14476. *J Hosp Infect.* 2021;111:180-183.
32. Teagle V, Clem DS, Yoon T. Virucidal Properties of Molecular Iodine Oral Rinse Against SARS-CoV-2. *Compend Contin Educ Dent.* 2022;43(2):e13-e16.
33. Tiong V, Hassandarvish P, Bakar SA, et al. The effectiveness of various gargle formulations and salt water against SARS-CoV-2. *Sci Rep.* 2021;11(1):20502.

34. Abdelalim AA, Mohamady AA, Elsayed RA, Elawady MA, Ghallab AF. Corticosteroid nasal spray for recovery of smell sensation in COVID-19 patients: A randomized controlled trial. *Am J Otolaryngol.* 2021;42(2):102884.
35. Chaudhary P, Melkonyan A, Meethil A, et al. Estimating salivary carriage of severe acute respiratory syndrome coronavirus 2 in nonsymptomatic people and efficacy of mouthrinse in reducing viral load: A randomized controlled trial. *J Am Dent Assoc.* 2021;152(11):903-908.
36. Burgos-Ramos E, Urbieta IR, Rodriguez D. Is hydrogen peroxide an effective mouthwash for reducing the viral load of SARS-CoV-2 in dental clinics? *Saudi Dent J.* 2022;34(3):237-242.
37. da Fonseca Orcina B, Vilhena FV, Cardoso de Oliveira R, et al. A Phthalocyanine Derivate Mouthwash to Gargling/Rinsing as an Option to Reduce Clinical Symptoms of COVID-19: Case Series. *Clin Cosmet Investig Dent.* 2021;13:47-50.
38. Domenico MBD, Collares K, Santos RBD, et al. Hydrogen peroxide as an auxiliary treatment for COVID-19 in Brazil: a randomized double-blind clinical trial. *Epidemiol Health.* 2021;43:e2021051.
39. Figueroa JM, Lombardo ME, Dogliotti A, et al. Efficacy of a Nasal Spray Containing Iota-Carrageenan in the Postexposure Prophylaxis of COVID-19 in Hospital Personnel Dedicated to Patients Care with COVID-19 Disease. *Int J Gen Med.* 2021;14:6277-6286.
40. Guenezan J, Garcia M, Strasters D, et al. Povidone Iodine Mouthwash, Gargle, and Nasal Spray to Reduce Nasopharyngeal Viral Load in Patients With COVID-19: A Randomized Clinical Trial. *JAMA Otolaryngol Head Neck Surg.* 2021;147(4):400-401.
41. Huang YH, Huang JT. Use of chlorhexidine to eradicate oropharyngeal SARS-CoV-2 in COVID-19 patients. *J Med Virol.* 2021;93(7):4370-4373.
42. Kasiri H, Rouhani N, Salehifar E, Ghazaeian M, Fallah S. Mometasone furoate nasal spray in the treatment of patients with COVID-19 olfactory dysfunction: A randomized, double blind clinical trial. *Int Immunopharmacol.* 2021;98:107871.
43. Arefin MK, Rumi S, Uddin A, et al. Virucidal effect of povidone iodine on COVID-19 in the nasopharynx: an open-label randomized clinical trial. *Indian J Otolaryngol Head Neck Surg.* 2021:1-5.
44. Khan MM, Parab SR, Paranjape M. Repurposing 0.5% povidone iodine solution in otorhinolaryngology practice in Covid 19 pandemic. *Am J Otolaryngol.* 2020;41(5):102618.
45. Saud Z, Tyrrell VJ, Zaragkoulias A, et al. The SARS-CoV2 envelope differs from host cells, exposes procoagulant lipids, and is disrupted in vivo by oral rinses. *J Lipid Res.* 2022;63(6):100208.
46. Avhad SKB, M.; Sachdev, S. S.; Save, S. S.; Kalra, D.; Kamala, D. N.. Comparison of effectiveness of chlorine dioxide mouthwash and chlorhexidine gluconate mouthwash in reduction of oral viral load in patients with covid-19. *Indian Journal of Public Health Research and Development* 2020;11(11):27-32.
47. Almanza-Reyes H, Moreno S, Plascencia-Lopez I, et al. Evaluation of silver nanoparticles for the prevention of SARS-CoV-2 infection in health workers: In vitro and in vivo. *PLoS One.* 2021;16(8):e0256401.
48. Aref ZF, Bazeed S, Hassan MH, et al. Clinical, Biochemical and Molecular Evaluations of Ivermectin Mucoadhesive Nanosuspension Nasal Spray in Reducing Upper Respiratory Symptoms of Mild COVID-19. *Int J Nanomedicine.* 2021;16:4063-4072.
49. Laferl H, Seitz T, Baier-Grabner S, et al. Evaluation of RT-qPCR of mouthwash and buccal swabs for detection of SARS-CoV-2 in children and adults. *Am J Infect Control.* 2022;50(2):176-181.

50. Michel W, Farber J, Dilas M, et al. A combined oro-nasopharyngeal swab is more sensitive than mouthwash in detecting SARS-CoV-2 by a high-throughput PCR assay. *Infection.* 2021;49(3):527-531.
51. Mora-Aguilera G, Martinez-Bustamante V, Acevedo-Sanchez G, et al. Surveillance Web System and Mouthwash-Saliva qPCR for Labor Ambulatory SARS-CoV-2 Detection and Prevention. *Int J Environ Res Public Health.* 2022;19(3).
52. Paull JRA, Luscombe CA, Castellarnau A, Heery GP, Bobardt MD, Gallay PA. Protective Effects of Astodrimer Sodium 1% Nasal Spray Formulation against SARS-CoV-2 Nasal Challenge in K18-hACE2 Mice. *Viruses.* 2021;13(8).
53. Errecalde J, Lifschitz A, Vecchioli G, et al. Safety and Pharmacokinetic Assessments of a Novel Ivermectin Nasal Spray Formulation in a Pig Model. *J Pharm Sci.* 2021;110(6):2501-2507.
54. Jain A, Grover V, Singh C, et al. Chlorhexidine: An effective anticovid mouth rinse. *J Indian Soc Periodontol.* 2021;25(1):86-88.
55. Bano-Polo M, Martinez-Gil L, Sanchez Del Pino MM, et al. Cetylpyridinium chloride promotes disaggregation of SARS-CoV-2 virus-like particles. *J Oral Microbiol.* 2022;14(1):2030094.
56. Bansal S, Jonsson CB, Taylor SL, et al. Iota-carrageenan and xylitol inhibit SARS-CoV-2 in Vero cell culture. *PLoS One.* 2021;16(11):e0259943.
57. Bentley K, Stanton RJ. Hydroxypropyl Methylcellulose-Based Nasal Sprays Effectively Inhibit In Vitro SARS-CoV-2 Infection and Spread. *Viruses.* 2021;13(12).
58. Bovard D, van der Toorn M, Schlage WK, et al. Iota-carrageenan extracted from red algae is a potent inhibitor of SARS-CoV-2 infection in reconstituted human airway epithelia. *Biochem Biophys Rep.* 2022;29:101187.
59. Haridas M, Sasidhar V, Nath P, Abhithaj J, Sabu A, Rammanohar P. Compounds of Citrus medica and Zingiber officinale for COVID-19 inhibition: in silico evidence for cues from Ayurveda. *Futur J Pharm Sci.* 2021;7(1):13.
60. Moakes RJA, Davies SP, Stamataki Z, Grover LM. Formulation of a Composite Nasal Spray Enabling Enhanced Surface Coverage and Prophylaxis of SARS-COV-2. *Adv Mater.* 2021;33(26):e2008304.
61. Morokutti-Kurz M, Froba M, Graf P, et al. Iota-carrageenan neutralizes SARS-CoV-2 and inhibits viral replication in vitro. *PLoS One.* 2021;16(2):e0237480.
62. Munoz-Basagoiti J, Perez-Zsolt D, Leon R, et al. Mouthwashes with CPC Reduce the Infectivity of SARS-CoV-2 Variants In Vitro. *J Dent Res.* 2021;100(11):1265-1272.
63. Paolacci S, Ergoren MC, De Forni D, et al. In vitro and clinical studies on the efficacy of alpha-cyclodextrin and hydroxytyrosol against SARS-CoV-2 infection. *Eur Rev Med Pharmacol Sci.* 2021;25(1 Suppl):81-89.
64. Rodriguez K, Saunier F, Rigaill J, et al. Evaluation of in vitro activity of copper gluconate against SARS-CoV-2 using confocal microscopy-based high content screening. *J Trace Elem Med Biol.* 2021;68:126818.
65. Sharad S, Kapur S. Indian Herb-Derived Phytoconstituent-Based Antiviral, Antimicrobial and Antifungal Formulation: An Oral Rinse Candidate for Oral Hygiene and the Potential Prevention of COVID-19 Outbreaks. *Pathogens.* 2021;10(9).
66. Tateyama-Makino R, Abe-Yutori M, Iwamoto T, et al. The inhibitory effects of toothpaste and mouthwash ingredients on the interaction between the SARS-CoV-2 spike protein and ACE2, and the protease activity of TMPRSS2 in vitro. *PLoS One.* 2021;16(9):e0257705.

67. Yadalam PK, Varatharajan K, Rajapandian K, et al. Antiviral Essential Oil Components Against SARS-CoV-2 in Pre-procedural Mouth Rinses for Dental Settings During COVID-19: A Computational Study. *Front Chem.* 2021;9:642026.
68. Rodriguez-Casanovas HJ, la Rosa M, Bello-Lemus Y, Rasperini G, Acosta-Hoyos AJ. Virucidal Activity of Different Mouthwashes Using a Novel Biochemical Assay. *Healthcare (Basel).* 2021;10(1).
69. Robinson TE, Moakes RJA, Grover LM. Low Acyl Gellan as an Excipient to Improve the Sprayability and Mucoadhesion of Iota Carrageenan in a Nasal Spray to Prevent Infection With SARS-CoV-2. *Front Med Technol.* 2021;3:687681.
70. Westover JB, Ferrer G, Vazquez H, Bethencourt-Mirabal A, Go CC. In Vitro Virucidal Effect of Intranasally Delivered Chlorpheniramine Maleate Compound Against Severe Acute Respiratory Syndrome Coronavirus 2. *Cureus.* 2020;12(9):e10501.
71. Mark L Cannon JBW, Reiner Bleher, Marcos A. Sanchez-Gonzalez, Gustavo Ferrer. In Vitro Analysis of the Anti-viral Potential of nasal spray constituents against SARS-CoV-2. *BioRxiv.* December 2020.
72. Nurul Azmawati Mohamed NB, Wan Shahida Wan Sulaiman, Zetti Zainol Rashid, Wong Kon Ken, Umi Kalsom Ali, Siti Norlia Othman, Muttaqillah Najihan Samat, Najma Kori, Petrick Periyasamy, Nor Azizan Zakaria, Agni Nhirmal Kumar Sugurmar, Nur Ezzaty Mohammad Kazmin, Cheong Xiong Khee, Siti Mariyam Saniman, Ilina Isahak. EARLY VIRAL CLEARANCE AMONG COVID-19 PATIENTS WHEN GARGLING WITH POVIDONE-IODINE AND ESSENTIAL OILS – A CLINICAL TRIAL. *medRvix.* Sep 2020.
73. Evelina Statkute AR, Valerie B O'Donnell, David W. Thomas, Richard J. Stanton. Brief Report: The Virucidal Efficacy of Oral Rinse Components Against SARS-CoV-2 In Vitro. *bioRxiv.* Nov 2020.
74. Xu C, Wang A, Hoskin ER, et al. Differential effects of antiseptic mouth rinses on SARS-CoV-2 infectivity in vitro. *bioRxiv.* 2020.
75. Buonavoglia A, Camero M, Lanave G, et al. Virucidal activity in vitro of mouthwashes against a feline coronavirus type II. *Oral Dis.* 2021.
76. A. Green GR, T. Tobery, C. Vincent, M. Barili, C. Jones. In vitro assessment of the virucidal activity of four mouthwashes containing Cetylpyridinium Chloride, ethanol, zinc and a mix of enzyme and proteins against a human coronavirus. In. *bioRxiv*2020.
77. Shet M, Hong R, Igo D, Cataldo M, Bhaskar S. In Vitro Evaluation of the Virucidal Activity of Different Povidone-Iodine Formulations Against Murine and Human Coronaviruses. *Infect Dis Ther.* 2021;10(4):2777-2790.
78. Balouch B, Vontela S, Yeakel H, Alnouri G, Sataloff RT. Role of Famotidine and Other Acid Reflux Medications for SARS-CoV-2: A Pilot Study. *J Voice.* 2021.
79. Frank S, Capriotti J, Brown SM, Tessema B. Povidone-Iodine Use in Sinonasal and Oral Cavities: A Review of Safety in the COVID-19 Era. *Ear Nose Throat J.* 2020;99(9):586-593.
80. Vergara-Buenaventura A, Castro-Ruiz C. Use of mouthwashes against COVID-19 in dentistry. *Br J Oral Maxillofac Surg.* 2020;58(8):924-927.
81. de Toledo Telles-Araujo G, Caminha RDG, Kallas MS, Sipahi AM, da Silva Santos PS. Potential mouth rinses and nasal sprays that reduce SARS-CoV-2 viral load: What we know so far? *Clinics (Sao Paulo).* 2020;75:e2328.
82. Gerlach M, Wolff S, Ludwig S, et al. Rapid SARS-CoV-2 inactivation by commonly available chemicals on inanimate surfaces. *J Hosp Infect.* 2020;106(3):633-634.
83. Carrouel F, Conte MP, Fisher J, et al. COVID-19: A Recommendation to Examine the Effect of Mouthrinses with beta-Cyclodextrin Combined with Citrox in Preventing Infection and Progression. *J Clin Med.* 2020;9(4).

84. Peng X, Xu X, Li Y, Cheng L, Zhou X, Ren B. Transmission routes of 2019-nCoV and controls in dental practice. *Int J Oral Sci.* 2020;12(1):9.
85. Pattanshetty S, Narayana A, Radhakrishnan R. Povidone-iodine gargle as a prophylactic intervention to interrupt the transmission of SARS-CoV-2. *Oral Dis.* 2021;27 Suppl 3:752-753.
